# Characterization and Antioxidant Activity Determination of Neutral and Acidic Polysaccharides from *Panax Ginseng* C. A. Meyer

**DOI:** 10.3390/molecules25040791

**Published:** 2020-02-12

**Authors:** Hyung Min Kim, Yanxue Song, Gyu Hwan Hyun, Nguyen Phuoc Long, Jeong Hill Park, Yves S.Y. Hsieh, Sung Won Kwon

**Affiliations:** 1 College of Pharmacy, Seoul National University, Seoul 08826, Korea; snuhmkim04@snu.ac.kr (H.M.K.); 2016-28995@snu.ac.kr (Y.S.); kinggury@snu.ac.kr (G.H.H.); phuoclong@snu.ac.kr (N.P.L.); hillpark@snu.ac.kr (J.H.P.); 2 Division of Glycoscience, Department of Chemistry, School of Engineering Sciences in Chemistry, Biotechnology and Health, Royal Institute of Technology (KTH), SE-106 91 Stockholm, Sweden; yvhsieh@kth.se; 3 Plant Genomics and Breeding Institute, Seoul National University, Seoul 08826, Korea

**Keywords:** *Panax ginseng*, acidic polysaccharide, neutral polysaccharide, *Caenorhabditis elegans*, antioxidant effect

## Abstract

*Panax ginseng* (*P. ginseng*) is the most widely consumed herbal plant in Asia and is well-known for its various pharmacological properties. Many studies have been devoted to this natural product. However, polysaccharide’s components of ginseng and their biological effects have not been widely studied. In this study, white ginseng neutral polysaccharide (WGNP) and white ginseng acidic polysaccharide (WGAP) fractions were purified from *P. ginseng* roots. The chemical properties of WGNP and WGAP were investigated using various chromatography and spectroscopy techniques, including high-performance gel permeation chromatography, Fourier-transform infrared spectroscopy, and high-performance liquid chromatography with an ultra-violet detector. The antioxidant, anti-radical, and hydrogen peroxide scavenging activities were evaluated in vitro and in vivo using *Caenorhabditis elegans* as the model organism. Our in vitro data by ABTS (2,2′-azino-bis-(3-ethylbenzothiazoline-6-sulfonic acid), reducing power, ferrous ion chelating, and hydroxyl radical scavenging activity suggested that the WGAP with significantly higher uronic acid content and higher molecular weight exhibits a much stronger antioxidant effect as compared to that of WGNP. Similar antioxidant activity of WGAP was also confirmed in vivo by evaluating internal reactive oxygen species (ROS) concentration and lipid peroxidation. In conclusion, WGAP may be used as a natural antioxidant with potent scavenging and metal chelation properties.

## 1. Introduction

Plant cell wall polysaccharides are natural biopolymers that serve as energy and dietary fiber sources and are essential biomolecules that are involved in critical biological functions related to plant growth and development [1,2]. These polysaccharides possess potential pharmacological properties, such as antioxidant, antitumor, antiviral, and immunomodulation activities that have been widely utilized. *Panax ginseng* C.A. Meyer (*P. ginseng*) is a perennial herb that belongs to the Araliaceae family and is cultivated in Korea, China, and Japan [3,4,5]. In traditional medicine, the plant extracts from *P. ginseng* have been used as tonics for the treatment of ischemic heart disease, common cold, obstructive pulmonary disease, and erectile dysfunction [6,7,8,9]. However, these effects were mostly from ginsenosides, which are the most widely studied compounds from ginseng. Therefore, other types of compounds from ginseng should be widely studied. Some of the previous studies have shown that ginseng polysaccharides are biologically active compounds with substantial anticancer and anti-hyperglycemic activities [10,11]. The antioxidant activities of ginseng polysaccharide have been reported, but the mechanism by which the structural components can lower the oxidative stress levels is not known. As far as we are concerned, there were no comparative studies of the acidic and neutral polysaccharide derived from white ginseng. Therefore, extensive studies of polysaccharide from white ginseng is in need.

Reactive oxygen species (ROS) are chemically active compounds that can cause cellular damage. Generally, ROS are produced as byproducts of cellular metabolism, mostly from the oxidative phosphorylation process that occurs in the mitochondrial electron transport chain [12]. ROS is essential in maintaining cellular homeostasis, but external stress factors, such as chemicals, can also lead to the production of ROS. An increased ROS production can incur damages to the lipids, proteins, and nucleic acids [13,14]. Oxidative stress accumulation can lead to metabolic disorders such as cancer, aging, inflammation, and neurodegeneration [15,16]. The cells have enzymatic antioxidative defense systems to compensate for the ROS concentration to maintain the equilibrium [17,18,19], including the enzymes superoxide dismutase (SOD), catalase (CAT), and glutathione peroxidase (GSH-Px) [20,21]. In addition, ascorbic acid and tocopherol are also important non-enzymatic antioxidants that can regulate the levels of ROS [22]. Recent studies have found that many compounds derived from the dietary fibers can scavenge ROS [23,24]. Moreover, the antioxidant effect is a key biological effect derived from many plant-based materials. Several studies emphasized antioxidant effects from different types of compounds [25,26]. The polysaccharides isolated from plants are shown to possess free radical scavenging activities, including the inhibition of lipid peroxidation and protein carbonylation [27,28].

In this study, neutral and acidic polysaccharides of ginseng were separated and purified using anion-exchange chromatography, and their chemical composition and antioxidant activities were examined thoroughly. Furthermore, we performed an in vivo study using *Caenorhabditis elegans* (*C. elegans*), which is a widely used model organism. As a result, the biological activities of ginseng polysaccharides were evaluated. In addition, the performance of acidic polysaccharide and neutral polysaccharide was compared. We found that the pectic polysaccharide fraction exhibited vigorous ROS scavenging activity, and may find the applications in different industrial sectors. 

## 2. Results and Discussion

### 2.1. Extraction and Isolation of Polysaccharide from Panax Ginseng

**Figure 1:**
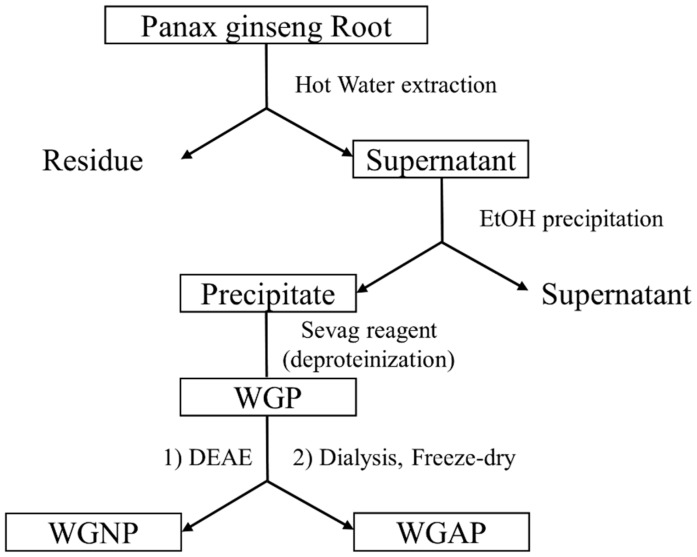
The flowchart of extraction of ginseng polysaccharide fractions.

Panax ginseng has been used as traditional medicine for a long time for the treatment of various diseases. Their pharmacological effect is well studied by many research groups which makes ginseng a valuable source in pharmaceutical industries. *Panax ginseng* has been studied intensively during the past several decades [29]. The efficacy and safety of ginseng have been demonstrated in clinical trials [6]. The effects of ginseng consist of various conditions in the central nervous system [30] and cardiovascular systems [8], among others. However, most studies on the biological effects of ginseng focused on ginsenosides. Therefore, this study set out to evaluate the pharmacological effects of ginseng polysaccharides. The crude extracts of the polysaccharides were prepared from the root of *P. ginseng* by water extraction and ethanol precipitation (Figure 1). The extracts were further purified by diethylaminoethyl (DEAE) cellulose anion-exchange column using water and a NaCl solution as eluent. After collection, these fractions were concentrated, dialyzed, and lyophilized to obtain a dried powder. As a result, two purified polysaccharides, white ginseng neutral polysaccharide (WGNP) and white ginseng acidic polysaccharide (WGAP) were obtained.

### 2.2. Chemical Compositions and Molecular Weight of WGNP and WGAP

The carbohydrate, protein, and the uronic acid content, as well as the molecular weights of WGNP and WGAP are listed in Table 1. There was a significant difference between WGNP and WGAP in terms of carbohydrate and uronic acid content. In particular, the uronic acid content of WGNP and WGAP was 0.8% and 25.7%, respectively, which suggests that WGNP is a neutral polysaccharide and WGAP is an acidic polysaccharide. The monosaccharide composition of WGNP and WGAP were analyzed (Table 2). WGNP was composed of mostly glucose (97.9%), suggesting the fraction is mainly composed of starch. Whereas the WGAP is mainly composed of mainly galactose, glucose, arabinose, and galacturonic acid with proportions of 24.4, 24.0, 18.1, and 32.2%, respectively, which indicates the presence of pectins with arabinogalactan side chains. The molecular weights of two polysaccharide fractions were analyzed to be 16.1–70.4 kDa for WGNP and 50.0–80.0 kDa for WGAP by high-performance gel permeation chromatography (HPGPC). Interestingly the acidic polysaccharides pectins had higher molecular weight as compared to the neutral polysaccharide starch.

### 2.3. Fourier-Transform Infrared Spectroscopy (FT-IR) Spectrum of WGNP and WGAP

**Figure 2:**
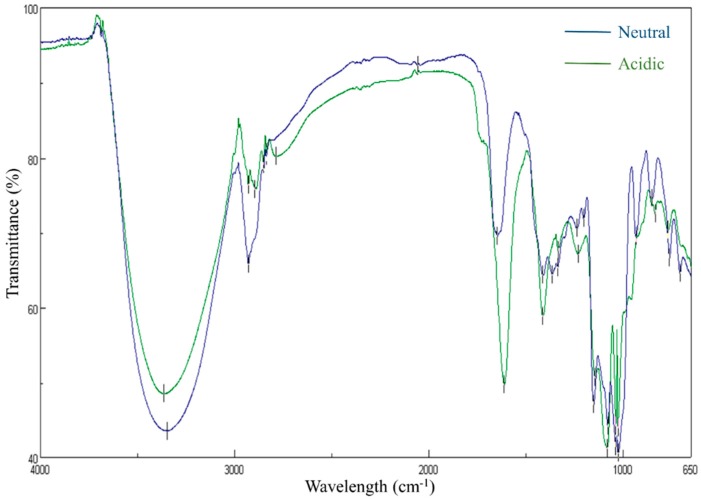
The FT-IR spectrum of WGNP (Neutral) and WGAP (Acidic).

Through FT-IR studies, functional groups of WGNP and WGAP were monitored within the range of 500–4000 cm^−1^ (Figure 2). Broadly stretched peak in the range of 3600–3000 cm^−1^ was attributed to a hydroxyl stretching vibration and a weak C-H stretching vibration was observed around 3000–2800 cm^−1^. The absorption peak near 1600 and 1400 cm^−1^ was attributed to the C=O asymmetric stretching vibration, while the peak around 1250 cm^−1^ of WGAP indicated vibration of the variable O-H angle, which represents acidic polysaccharide. Furthermore, a peak near 1050 indicated glycosidic bonds [31,32].

### 2.4. In vitro Antioxidant Activities of Polysaccharides

After isolation and characterization of WGNP and WGAP, we hypothesized that they could exhibit antioxidant effects. Thus, we first investigated the antioxidant activities by ABTS (2,2′-azino-bis-(3-ethylbenzothiazoline-6-sulfonic acid) radical scavenging activity, reducing power, ferrous ion chelating activity, and hydroxy radical scavenging activity. ABTS is a widely used method to assess antioxidant effects from natural products. It is a colorimetric assay by ABTS cation radical formation [33]. Reducing power is determined by compounds with reduction potential. It reacts with potassium ferricyanide and results in potassium ferrocyanide. Then, it forms a complex with ferric chloride, which shows absorbance at 700 nm [34]. Next, chelating activities of antioxidants were evaluated by inhibition in the formation of Fe^2+^–ferrozine complex, which has an absorbance at 562 nm [35]. EDTA was used as a positive control in this assay. Lastly, hydroxyl radical scavenging activity was performed. Hydroxy radicals can penetrate cell membranes and damage essential elements, such as proteins, lipids, and DNA. Therefore, scavenging these compounds is an essential defense mechanism in living organisms [32]. The hydroxyl radical scavenging activities were determined by measuring the increase in the formation of a Fe^2+^-1, 10-phenanthroline complex. H_2_O_2_ will oxidize Fe^2+^ to Fe^3+^, which makes it hard to form a red-orange complex with 1, 10-phenanthroline. When the antioxidant compound is added, conversion of the ferrous ion is decreased resulting in an increased concentration of a 1, 10-phenanthroline complex. Therefore, after adding white ginseng polysaccharides, the absorbance of red-orange complex was monitored at 510 nm. Ascorbic acid was used as a positive control for our in vitro studies since it is a representative low-molecular-weight antioxidant that exhibits an apparent antioxidant effect and could be purchased easily [32,36].

#### 2.4.1. ABTS Radical Scavenging Activity

**Figure 3:**
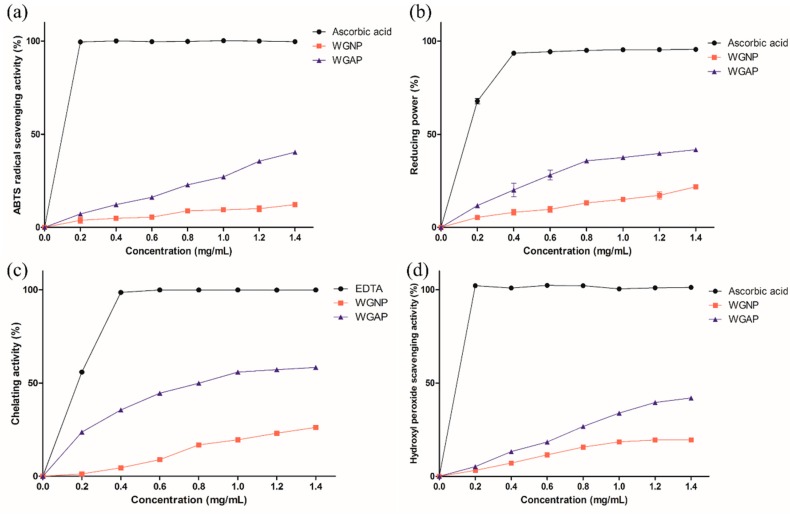
Antioxidant activities of WGNP and WGAP on (**a**) ABTS (2,2′-azino-bis-(3-ethylbenzothiazoline-6-sulfonic acid) radical scavenging, (**b**) reducing power, (**c**) ferrous chelating, and (**d**) hydroxyl peroxide scavenging activities.

ABTS forms stable radicals when it reacts with potassium persulfate, which has the absorbance maxima at wavelength 734 nm. The addition of antioxidants decreases the absorbance at 734 nm since the antioxidants inhibit the formation of ABTS radicals, thus decreasing its concentration in the solution [37]. Figure 3a shows the scavenging effects of WGNP and WGAP with ascorbic acid as a positive control. Ascorbic acid showed substantial scavenging activity even at a low concentration. On the other hand, scavenging activities of samples increased in a concentration-dependent manner. WGAP showed higher scavenging activity as compared to WGNP. At a concentration of 1.40 mg/mL, WGNP and WGAP showed 13.03% and 40.94% scavenging effects, respectively. Furthermore, EC_50_ was calculated as 9.69 and 2.45 mg/mL for WGNP and WGAP, respectively. These results suggest that WGAP is more effective at scavenging ABTS radicals.

#### 2.4.2. Reducing Power

The samples that have reducing power can provide electrons to have scavenging effects on the free radicals. Thus, by measuring the reducing power, the antioxidant effect can be evaluated. In these experiments, we assessed the amount of a ferrous-ferric ion complex, which can be measured by absorbance at 700 nm. Figure 3b shows the reducing power of WGNP and WGAP as compared with positive control ascorbic acid. The reducing power of two samples and ascorbic acid increased gradually as the concentration increased. Moreover, the reducing power of WGAP was higher than WGNP. At 1.40 mg/mL, the reducing power of WGNP and WGAP was 21.78% and 41.68%, respectively. In addition, EC_50_ was calculated as 5.33 and 1.68 mg/mL for WGNP and WGAP, respectively. Our results suggest that WGAP exhibited a higher effect on reducing power, which showed similar results to the other antioxidant activities.

#### 2.4.3. Ferrous Ion Chelating Activity

The chelating activities of antioxidants were evaluated by a ferrous ion chelating activity. The results of WGNP and WGAP are shown in Figure 3c using EDTA as a positive control. The chelating effect was increased in a dose-dependent manner and was 26.23% and 58.34% for WGNP and WGAP at 1.40 mg/mL, respectively. Furthermore, EC_50_ was calculated as 4.31 and 0.81 mg/mL for WGNP and WGAP, respectively. WGAP had a higher chelating activity, which could be resulted from the higher carboxylic group content, as indicated by the uronic acid content.

#### 2.4.4. Hydroxyl Radical Scavenging Activities

The hydroxyl radical scavenging activities of WGNP and WGAP are shown in Figure 3d. The hydroxyl radical scavenging activities were increased in proportion to the concentration, which was 19.83% and 41.06% for WGNP and WGAP at 1.40 mg/mL, respectively. Moreover, EC_50_ was calculated as 4.94 and 2.09 mg/mL for WGNP and WGAP, respectively. As a result, WGP showed significant hydroxyl radical scavenging activities, and these effects might be related to the presence of the active hydroxyl group in the polysaccharide.

### 2.5. Antioxidant Activity of Polysaccharide on C. elegans

The above section described different aspects of the antioxidant effects of WGNP and WGAP in vitro. Nevertheless, it would be better to demonstrate the potency of antioxidant effects using living species. In this study, we selected *C. elegans* to evaluate antioxidant activity in the living organism. *C. elegans* is a widely used model organism in studying diseases and biological assays. Its advantages are a short life cycle, well-characterized genome, and easy maintenance, which make a valuable model for scientific studies [19]. Applying this model organism will help to understand the effect of polysaccharide on the living organism. We applied the antioxidant effect by evaluating oxidative stress and lipid peroxidation in *C. elegans*.

#### 2.5.1. Lethality Assays of Polysaccharide on *C. elegans*

To find the optimal concentration of polysaccharide in *C. elegans* experiments, *C. elegans* was exposed to WGNP and WGAP at varying concentrations. A concentration from 0 mg/mL to 1.00 mg/mL did not affect their survival rate. However, when the polysaccharides concentration was greater 1.00 mg/mL, the survival rate was significantly reduced in the preliminary tests (data not shown). Therefore, we selected 1.00 mg/mL as an exposure condition for *C. elegans,* which did not have toxic effects regarding lethality.

#### 2.5.2. Antioxidant Effect of WGP on Oxidative Stress-Induced *C. elegans*

Reactive oxygen species (ROS) are highly reactive molecules, such as hydrogen peroxide, hydroxyl radical, and superoxide anion. ROS are usually produced by cellular aerobic metabolism and can increase dramatically when the organisms are exposed to environmental stress. Several lines of evidence have suggested that polysaccharides derived from different types of plants that exhibit antioxidant effects [38,39]. The results from in vitro experiments suggested that WGP might possess antioxidant effects. Thus, we further examined the anti-oxidative capacity of WGP using oxidative stress-induced *C. elegans* as an in vivo model. In particular, a non-fluorescent compound, 2,7-dichlorodihydrofluorescein diacetate (H_2_DCFDA), was used for the evaluation of intracellular ROS. This reagent can penetrate the cell membrane and is transformed into a fluorescence compound dichlorofluorescein (DCF) when it reacts with ROS. Thus, intracellular ROS can be measured by monitoring the fluorescence at 485/535 nm.

**Figure 4:**
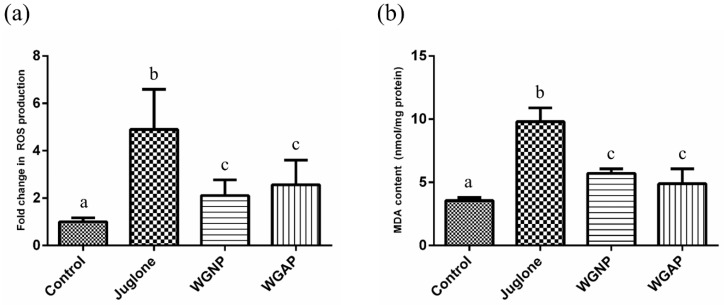
Antioxidant activities of WGNP and WGAP on *Caenorhabditis elegans*. (**a**) Fold change in reactive oxygen species (ROS) production, (**b**) malondialdehyde (MDA) content. All data are expressed as mean ± S.D. The different letters represent a statistical difference at *p* < 0.05 among the groups.

In this in vivo experiment, juglone was used for generating the ROS. The effects of WGNP and WGAP on oxidative stress in *C. elegans* were investigated. Our study showed *C. elegans* exposed to juglone had increased ROS levels, but when supplemented with WGNP and WGAP, we found a decrease in ROS levels in the WGNP and WGAP treated worms as compared to the group of worms treated with juglone only (Figure 4). The data suggested that both WGNP and WGAP had a protective effect against oxidative stress in vivo. In addition, we found that the WGAP treatment caused a much more significant reduction in internal ROS levels as compared to the WGNP treatment, suggesting that WGAP had a stronger protective effect against oxidative stress. The higher uronic acid content and higher molecular weight of the pectic arabinogalactan side chains of WGAP might be responsible for this observation. However, the exact mechanism of the protective role of WGAP should be further explored.

#### 2.5.3. Lipid Peroxidation Assay

Lipid peroxidation assay is a standard method to evaluate the effects of oxidation on organisms. It involves the oxidative degeneration of lipids, which is induced by ROS. As a result, several aldehydes are formed as the end products. These compounds can have a harmful effect on the organism. Among them, malondialdehyde (MDA) is considered a significant compound for causing oxidative stress. MDA is widely used to evaluate oxidative stress effects on lipids. In this study, the thiobarbituric acid (TBA) method was applied to measure the amount of MDA. TBA can react with MDA and form thiobarbituric acid reactive substances (TBARS), which have absorbance at 532 nm. Figure 4 shows that the addition of polysaccharide fraction (1.00 mg/mL) to juglone could decrease the levels of MDA as compared to the juglone group. This indicates that the supplementation of polysaccharide acts as a defense mechanism towards oxidative stress in *C. elegans*. However, unlike other results, WGNP showed similar antioxidant activities as WGAP. For in vivo experiments using model organisms, several factors such as metabolism, bioavailability, and the digestion ability of compounds affected antioxidant activities [40]. These factors may affect the potency of the antioxidant activities from WGNP and WGAP. Therefore, detailed mechanisms of the biological properties of polysaccharide should be further studied.

## 3. Materials and Methods

### 3.1. Materials and Reagent

Plants were purchased from a local ginseng market in Seoul, Korea. The samples were washed, dried, and pulverized into a powder. The chemical reagents, sulfuric acid, phenol, ascorbic acid, 2,2′-azino-bis-(3-ethylbenzothiazoline-6-sulfonic acid) (ABTS), potassium persulfate, potassium ferricyanide, ferrozine, ferric chloride, and phenanthroline, were purchased from Sigma Aldrich (St. Louis, MO, USA). Solvents, such as ethanol, ether, and chloroform, were purchased from J. T. Baker (Phillipsburg, NJ, USA). Agar, bactopeptone, and LB broth were purchased from Becton Dickson (Sparks, MD, USA).

### 3.2. Extraction and Purification of Polysaccharides

The crude polysaccharide was extracted from dried root powder by boiling in water at 100 °C for 4 h. The supernatant was collected, and the insoluble residues underwent the same extraction procedure twice before the extracts were combined and concentrated using an evaporator. The polysaccharides were precipitated by the addition of 4 volumes of ethanol. The precipitates were then washed with ethanol and ether before being dried under a stream of nitrogen gas. The crude ginseng polysaccharide was treated with a Sevag reagent (1:4 butanol:chloroform) to remove proteins [41]. After that, white ginseng polysaccharide (WGP) was obtained by completely drying the solvent using nitrogen gas.

WGP (5 g) was dissolved in 100 mL of distilled water and loaded on a DEAE Cellulose column (2.0 × 30 cm). It was eluted with distilled water and 0.5 M NaCl, sequentially. The fractions were collected at consistent intervals and the sugar concentration was monitored by phenol-sulfuric acid assay. The two significant fractions were collected, dialyzed, and concentrated to obtain neutral (WGPN) and acidic polysaccharide (WGPA) fractions. These were dissolved again in the desired concentration for further analysis, as described below.

### 3.3. Characterization of WGPN and WGPA

#### 3.3.1. Chemical Properties

The total carbohydrate content was analyzed by the phenol-sulfuric acid colorimetric method using glucose as the reference standard [42]. The uronic acid content was determined by the *m*-hydroxydiphenyl method using galacturonic acid as the reference standard [43]. The protein content was quantified by bicinchoninic acid assay (BCA assay) (Thermo Fisher Scientific, Waltham, MA, USA) using bovine serum albumin as the reference standard [44]. The average molecular weight was evaluated by HPGPC using a set of pullulan standards for the construction of the molecular weight distribution curve.

#### 3.3.2. Fourier-Transform Infrared Spectroscopy (FT-IR)

The FT-IR spectra for WGNP and WGAP were compared using the FT-IR spectrophotometer (FT/IR-4200, Jasco, Tokyo, Japan). The spectral range of 4000–500 cm^−1^ was analyzed.

#### 3.3.3. Monosaccharide Composition

The monosaccharide composition analysis was performed by HPLC using the 1-phenyl-3-methyl-5-pyrazolone (PMP) derivatization method, as previously described [45]. Briefly, 10 mg of the sample was hydrolyzed in 10 mL of 1 M hydrochloric acid in methanol at 80 °C for 16 h under a nitrogen gas flow. Then, 10 mL of 2 M trifluoroacetic acid was added, followed by incubation at 120 °C for 1 h. One milliliter of the sample was transferred to a separate vial and dried under a stream of nitrogen gas. Sodium hydroxide solution (0.3 M, 0.5 mL) was added to dissolve the dried material, which was mixed with 0.5 M PMP (0.5 mL), before being incubated at 70 °C for 30 min. Following this, 0.3 M hydrochloric acid was added to neutralize the pH, and then chloroform was added; the mixture was vortexed and the aqueous layer was collected. The same procedure was repeated three times; then the mixture was filtered through a 0.45 μm filter, and the PMP-derivatized sugars were analyzed by HPLC.

For the analysis of PMP derivatives, the Agilent 1200 series HPLC system equipped with a diode array detector was used. The PMP sugars were separated using the Eclipse C18 column (4.6 × 150 mm, 3.5 μm, Agilent, Santa Clara, CA, USA). The mobile phase consisted of 20% acetonitrile in 20 mM phosphate buffer (Mobile phase A) and 30% acetonitrile in 20 mM phosphate buffer (Mobile phase B). The flow rate was 1 mL/min at a temperature of 30 °C, and the injection volume was 10 μL. Each monosaccharide was quantified using standards at a detection wavelength of 245 nm.

### 3.4. In vitro Antioxidant Activities of Polysaccharides

#### 3.4.1. ABTS Radical Scavenging Activity

ABTS (2,2′-azino-bis-(3-ethylbenzothiazoline-6-sulfonic acid) radical scavenging activity was adopted to evaluate the antioxidant activities of ginseng polysaccharides [46]. ABTS solution (7 mM) was prepared with 2.45 mM potassium persulfate and was incubated at room temperature in the dark for 16 h to generate ABTS radicals. Then, the solution was diluted with 0.01 M PBS buffer (pH 7.4) until the absorbance reached approximately 0.7 at 734 nm. The optimized ABTS solution (1 mL) was added to 0.5 mL solution of each polysaccharide at various concentrations (0–1.40 mg/mL). The reaction mixture was incubated at room temperature for 6 min, in the dark. The absorbance of the mixture was analyzed at 734 nm using ascorbic acid as the positive control. The ABTS scavenging activity was calculated as follows:

(1) $$ABTS\;Scavenging\;activity\;\left(\%\right)=\frac{\left(1-As\right)}{Ac}\times 100\;\left(\%\right),$$

where As is the absorbance of the sample and Ac is the absorbance of the blank.

#### 3.4.2. Reducing Power Method

Reducing the power of the polysaccharides was determined by a method reported previously [34]. In brief, each WGP was dissolved in water at varying concentrations (0–1.40 mg/mL). To the 1 mL of polysaccharide solution, 2.5 mL of 0.2 M phosphate buffer and 2.5 mL of 1% potassium ferricyanide were added, followed by incubation at 50 °C for 20 min. After incubation, 2.5 mL of 10% trichloroacetic acid and 0.5 mL of 0.1% ferric chloride were added. Next, the mixture was vortexed. The absorbance of the mixture was measured at 700 nm. The reducing power was calculated as follows:

(2) $$Reducing\;power\;\left(\%\right)=\frac{1-As}{Ac}\times 100\;\left(\%\right),$$

where As is the absorbance of the sample and Ac is the absorbance of the blank.

#### 3.4.3. Ferrous Ion Chelating Activity

The ferrous ion chelating activity was measured by following the previously described method with a slight modification [35]. Briefly, 0.1 mL polysaccharide sample was added to 0.5 mL ferrous chloride (0.2 mM) and 0.2 mL ferrozine (5 mM), followed by incubation at room temperature for 10 min. Then, the absorbance was measured at 562 nm, using EDTA as the positive control. The ferrous ion chelating activity was calculated as follows:

(3) $$Ferrous\;ion\;chelating\;activity\;\left(\%\right)=\frac{1-As}{Ac}\;\times 100\;\left(\%\right),$$

where As is the absorbance of the sample and Ac is the absorbance of the blank.

#### 3.4.4. Hydroxyl Radical Scavenging Activity

Hydroxyl radical scavenging activity was measured as previously described for 1,10-phenanthroline-Fe^2+^ oxidative assay [47]. First, 1 mL of FeSO_4_ and 1.5 mL of the polysaccharide solution at varying concentrations were mixed. H_2_O_2_ solution was added to the mixture, followed by incubation at room temperature for a few minutes. Then, 1 mL of 1, 10-phenanthroline was added, and the mixture was incubated at room temperature for 10 min. After incubation, the absorbance was measured at 510 nm and the hydroxyl radical scavenging activity was calculated as follows:

(4) $$Hydroxyl\;radical\;scavenging\;activity\;\left(\%\right)=\frac{As}{Ac}\times 100\;\left(\%\right),$$

where As is the absorbance of the sample and Ac is the absorbance of the blank.

### 3.5. Antioxidant Activity of Polysaccharide on C. elegans

#### 3.5.1. *C. elegans* Strain and Culture Condition

Wild type *C. elegans* (N2) were obtained from the Caenorhabditis Genetic Center (CGC, University of Minnesota, Minneapolis, MN, USA). *C. elegans* were maintained at 20 °C on nematode growth media (NGM) with *Escherichia coli* OP50 as a food source. Age synchronized worms were obtained by treating gravid nematodes with a bleach solution that dissolves gravid hermaphrodites without damaging their embryos [48]. With these age-synchronized worms, antioxidant assays were performed.

#### 3.5.2. Lethality Assay

Lethality assay was performed to optimize the treatment concentration of the polysaccharides. The L4 stage worms were exposed to varying concentrations of polysaccharide solutions for 24 h, and then the number of dead worms was counted. They were considered dead if they were not responding to stimuli given by using a platinum wire [49].

#### 3.5.3. Experimental Design

To investigate antioxidant activities in *C. elegans*, a ROS generator, juglone, was applied to induce oxidative stress in *C. elegans*. The synchronized worms were grown until the L4 stage on NGM plates with 50 μM 2′-Deoxy-5-fluorouridine (FuDR). Then, the worms were transferred to NGM containing 1.00 mg/mL polysaccharide and 40 μM juglone, and then stored at 20 °C for 24 h. These worms were collected and utilized for evaluating the antioxidant activities of polysaccharides.

#### 3.5.4. Intracellular ROS Assay

ROS production assay was performed based on the previously reported method [50,51]. After exposure to polysaccharides, approximately 1,000 worms were transferred to a transparent bottom black 96-well plate (Thermo Fisher Scientific, Rochester, NY, USA). To evaluate the intracellular ROS levels, 100 μM H_2_DCF-DA was added to the worms. The fluorescence was measured at 485/535 nm by a SpectraMax M5 multi-plate reader (Molecular Devices, San Jose, CA, USA). The protein content was quantified by the BCA assay for normalization. 

#### 3.5.5. Lipid Peroxidation Assay

The lipid peroxide levels were determined by the thiobarbituric acid colorimetric method for the measurement of malondialdehyde (MDA) content [52]. First, the collected worms (approximately 1,000 worms) were homogenized by ultrasonication. Then, 20% trichloroacetic acid and 2-thiobarbituric acid were added to the *C. elegans* homogenates. After vortexing, the samples were incubated at 100 °C for 60 min. The samples were cooled to room temperature and then centrifuged. The absorbance of the supernatant was measured at 532 nm to investigate the amount of thiobarbituric acid reactive substance (TBARS). The concentration of TBARS was calculated based on the molar extinction coefficient of 1.56 × 10^5^ M^−1^ cm^−1^.

### 3.6. Statistical Analysis

The acquired data were analyzed by one-way analysis of variance (ANOVA) and a *p*-value of < 0.05 was considered significant. The results are expressed as mean ± standard deviation (S.D.)

## 4. Conclusions

In this study, neutral polysaccharide and acidic polysaccharide fractions were obtained from *P. ginseng* C. A. Meyer. WGNP and WGAP were found to have different monosaccharide compositions and molecular weights. The content of uronic acid differed to a great extent, which was also reflected in the composition of monosaccharide. The in vitro antioxidant effects test revealed that WGAP had stronger protective effects than WGNP in a dose-dependent manner. The higher antioxidant activities of WGAP could be derived from the differences in the composition of polysaccharides. Furthermore, our study suggests that WGAP had higher potential as an antioxidant agent in preventing the worm against the oxidative stress. The potential of the white ginseng polysaccharide should be studied further. The elucidation of the detailed structure–activity relation of WGAP for oxidative stress prevention may have beneficial implications in cosmetics, food, and pharmaceutical industries. 

## Figures and Tables

**Table 1:** Chemical composition and average molecular weight of white ginseng neutral polysaccharide (WGNP) and white ginseng acidic polysaccharide (WGAP).

Sample	WGNP	WGAP
Carbohydrate (%)	77.4 ± 0.6	28.2 ± 1.8
Uronic Acid (%)	0.8 ± 0.1	25.7 ± 0.8
Protein (%)	1.8 ± 0.2	5.1 ± 0.1
Molecular Weight (kDa)	16.1–70.4	50.0–80.0

**Table 2:** Chemical composition of WGNP and WGAP.

Samples	Monosaccharide Composition (%)						
	Galactose	Glucose	Arabinose	Rhamnose	Mannose	GalA	GluA
WGNP	1.1	97.9	1.0	-	-	-	-
WGAP	24.4	24.0	18.1	-	-	32.2	1.3

GalA: galacturonic acid. GluA: glucuronic acid.

## Abbreviations

WGAP	White ginseng acidic polysaccharide
WGNP	White ginseng neutral polysaccharide
ROS	Reactive oxygen species
SOD	Superoxide dismutase
CAT	Catalase
GSH-Px	Glutathione peroxidase
ABTS	2-2′-azino-bis-(3-ethylbenzothiazoline-6-sulfonic acid)
WGP	White ginseng polysaccharide
DEAE	Diethylaminoethyl
BCA	Bicinchoninic acid
HPGPC	High-performance gel permeation chromatography
FT-IR	Fourier-transform infrared spectroscopy
PMP	1-phenyl-3-methyl-5-pyrazolone
NGM	Nematode growth media
FuDR	2′-Deoxy-5-fluorouridine
MDA	Malondialdehyde
TBARS	Thiobarbituric acid reactive substance
H_2_DCFDA	2,7-Dichlorodihydrofluorescein diacetate
